# Allyl 4-hydroxy­phenyl carbonate

**DOI:** 10.1107/S1600536809022387

**Published:** 2009-06-17

**Authors:** Víctor Hugo Flores Ahuactzin, Delia López, Sylvain Bernès

**Affiliations:** a Facultad de Ciencias Químicas, Universidad Autónoma de Puebla, Boulevard 14 Sur, Col. San Manuel, 72570 Puebla, Pue., Mexico; b DEP Facultad de Ciencias Químicas, UANL, Guerrero y Progreso S/N, Col. Treviño, 64570 Monterrey, NL, Mexico

## Abstract

The title mol­ecule, C_10_H_10_O_4_, is a functionalized carbonate used in the synthetic route to organic glasses. The central CH fragment of the allyl group is disordered over two positions, with occupancies in a 0.758 (10):0.242 (10)ratio. This disorder reflects the torsional flexibility of the oxygen–allyl group, although both disordered parts present the expected anti­clinal conformation, with O—CH_2_—CH=CH_2_ torsion angles of −111 (2) and 119.1 (4)°. The crystal structure is based on chains parallel to [010], formed by O⋯H—O hydrogen bonds involving hydroxyl and carbonyl groups as donors and acceptors, respectively. The mol­ecular packing is further stabilized by two weak C—H⋯π contacts from the benzene ring of the asymmetric unit with two benzene rings of neighboring mol­ecules.

## Related literature

The crystal structures of two allyl carbonates have been reported to date, see: Michelet *et al.* (2003 ▶); Burns & Forsyth (2008 ▶). For the use of allyl ester and allyl carbonate derivatives as precursors for organic glasses, see: Herrera *et al.* (2003 ▶); Herrera (2006 ▶).
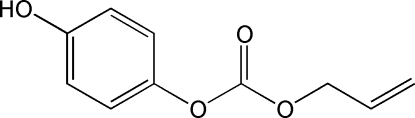


## Experimental

### 

#### Crystal data

C_10_H_10_O_4_
*M* *_r_* = 194.18
Monoclinic, 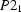

*a* = 5.8148 (7) Å
*b* = 7.5413 (11) Å
*c* = 11.4499 (17) Å
β = 93.515 (10)°
*V* = 501.15 (12) Å^3^
*Z* = 2
Mo *K*α radiation
μ = 0.10 mm^−1^
*T* = 298 K
0.60 × 0.30 × 0.24 mm

#### Data collection

Bruker P4 diffractometer
Absorption correction: none
2559 measured reflections
1233 independent reflections
1042 reflections with *I* > 2σ(*I*)
*R*_int_ = 0.025
3 standard reflections every 97 reflections intensity decay: 0.5%

#### Refinement

*R*[*F* ^2^ > 2σ(*F* ^2^)] = 0.037
*wR*(*F* ^2^) = 0.109
*S* = 1.06
1233 reflections
137 parameters
2 restraints
H-atom parameters constrained
Δρ_max_ = 0.10 e Å^−3^
Δρ_min_ = −0.17 e Å^−3^

### 

Data collection: *XSCANS* (Siemens, 1996 ▶); cell refinement: *XSCANS*; data reduction: *XSCANS*; program(s) used to solve structure: *SHELXS97* (Sheldrick, 2008 ▶); program(s) used to refine structure: *SHELXL97* (Sheldrick, 2008 ▶); molecular graphics: *Mercury* (Macrae *et al.*, 2006 ▶); software used to prepare material for publication: *SHELXL97*.

## Supplementary Material

Crystal structure: contains datablocks I, global. DOI: 10.1107/S1600536809022387/bq2136sup1.cif

Structure factors: contains datablocks I. DOI: 10.1107/S1600536809022387/bq2136Isup2.hkl

Additional supplementary materials:  crystallographic information; 3D view; checkCIF report

## Figures and Tables

**Table 1:** Hydrogen-bond geometry (Å, °)

*D*—H⋯*A*	*D*—H	H⋯*A*	*D*⋯*A*	*D*—H⋯*A*
O14—H14⋯O6^i^	0.88	1.91	2.762 (2)	160
C10—H10*A*⋯*Cg*^i^	0.93	2.90	3.612 (2)	135
C13—H13*A*⋯*Cg*^ii^	0.93	2.81	3.513 (3)	133

Symmetry codes: (i) 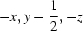; (ii) 
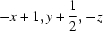
. *Cg* is the centroid of the benzene ring.

**Fig. 1.**
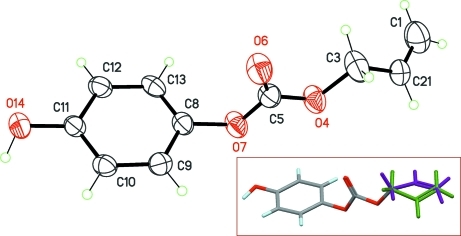
The structure of the title compound with displacement ellipsoids at the 30% probability level. C22 (minor part of the disorder) has been omitted for clarity. The inset shows the complete structure. Colour code: green for C21-disordered part; purple for the C22-disordered part.

**Fig. 2.**
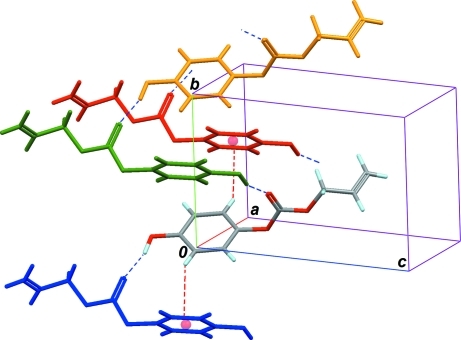
A part of the crystal structure of the title compound, including hydrogen bonds depicted with blue dashed lines, and C—H···π contacts from the asymmetric unit, represented as red dashed lines. Colour code for symmetry-related molecules: blue -x, -1/2+y, -z; green -x, 1/2+y, -z; red 1-x, 1/2+y, -z; gold x, 1+y, z.

## supplementary crystallographic information

### Comment

We have synthesized some functionalized aromatic allyl ester (Herrera *et al.*, 2003) and allyl carbonate compounds (Herrera, 2006) as intermediates in the preparation of new diallylcarbonate monomers, which are potential precursors of organic glasses. One representative example is the title molecule, which includes phenol and allyl functional groups as substitutents of the carbonate core (Fig. 1).

The allyl group presents a degree of flexibility, resulting in two disordered positions for the methylene group, C21 and C22, with site occupation factors 0.758 (10) and 0.242 (10), respectively (Fig. 1, inset). Regardless of the position for the CH_2_ fragment, *O*—allyl chain displays the expected *anticlinal* conformation, characterized by torsion angles O—CH_2_—CH═CH_2_ of -111 (2)° and 119.1 (4)°. A similar disorder was previously observed for a closely related phenol allylic derivative, allyl-4-hydroxybenzoate (Herrera *et al.*, 2003). To date, only two acyclic allyl carbonate systems have been *X* ray characterized, both with non disordered allyl groups. The *anticlinal* conformation has been stabilized in the first molecule, with *O*—allyl torsion angle of -131.4° (Michelet *et al.*, 2003), while in the most recent report, a *synperiplanar O*—allyl group is observed, the torsion angle being 9.7° (Burns & Forsyth, 2008). This very limited set of structurally characterized allyl carbonate derivatives does not allow to determine the factors influencing the stable conformation for these molecules in the solid state. In contrast, a number of allyl ester derivatives have been X-ray characterized, with conformations restricted to two attractors (Herrera *et al.*, 2003).

The crystal structure of the title compound is based on chains running along [010], formed through O···H—O bonds, where carbonyl functionalities act as acceptor and hydroxyl functionalities as donor groups. Moreover, two aryl C—H groups of the benzene ring of the asymmetric unit form stabilizing C—H···π contacts with the centroids of two symmetry-related benzene rings (Fig. 2).

### Experimental

Allyl 4-hydroxyphenyl-carbonate was synthesized by reacting hydroquinone and allylchloroformate in the presence of NaOH at 273 K for 2 h., affording a white powder (Yield: 43%). After recrystallization from a mixture of methylene chloride and hexane (7:3), colourless crystals were obtained. Anal. calcd for C_10_H_10_O_4_: C 61.85, H 5.19; found: C 61.85, H 5.25.

### Refinement

Allylic CH group was found to be disordered over two sites, C21 and C22, and occupancies were refined with the sum constrained to unity. In order to get a sensible geometry for the minor part, bond length C3—C22 was restrained to 1.480 (15)Å. C-bonded H atoms were placed in idealized positions and refined as riding to their carrier C atoms, with bond lengths fixed to 0.93Å (aromatic and allylic H atoms) or 0.97Å (methylene CH_2_). Hydroxyl H atom H14 was found in a difference map and refined as riding to O14, with the O—H bond length fixed to the found value, 0.883Å. Isotropic displacement parameters for H atoms were calculated as *U*_iso_(H) = 1.2*U*_eq_(carrier atom) for C-bonded H atoms and *U*_iso_(H) = 1.5*U*_eq_(O14) for H14. Measured Friedel pairs were merged in the final refinement.

### Crystal data

**Table:**

C_10_H_10_O_4_	*F*(000) = 204
*M**_r_* = 194.18	*D*_x_ = 1.287 Mg m^−^^3^
Monoclinic, *P*2_1_	Melting point: 337 K
Hall symbol: P 2yb	Mo *K*α radiation, λ = 0.71073 Å
*a* = 5.8148 (7) Å	Cell parameters from 52 reflections
*b* = 7.5413 (11) Å	θ = 4.7–13.6°
*c* = 11.4499 (17) Å	µ = 0.10 mm^−^^1^
β = 93.515 (10)°	*T* = 298 K
*V* = 501.15 (12) Å^3^	Prism, colorless
*Z* = 2	0.60 × 0.30 × 0.24 mm

### Data collection

**Table:**

Bruker P4 diffractometer	*R*_int_ = 0.025
Radiation source: fine-focus sealed tube	θ_max_ = 27.5°, θ_min_ = 3.2°
graphite	*h* = −7→5
2θ/ω scans	*k* = −9→1
2559 measured reflections	*l* = −14→14
1233 independent reflections	3 standard reflections every 97 reflections
1042 reflections with *I* > 2σ(*I*)	intensity decay: 0.5%

### Refinement

**Table:**

Refinement on *F*^2^	Primary atom site location: structure-invariant direct methods
Least-squares matrix: full	Secondary atom site location: difference Fourier map
*R*[*F*^2^ > 2σ(*F*^2^)] = 0.037	Hydrogen site location: inferred from neighbouring sites
*wR*(*F*^2^) = 0.109	H-atom parameters constrained
*S* = 1.06	*w* = 1/[σ^2^(*F*_o_^2^) + (0.0619*P*)^2^ + 0.0247*P*] where *P* = (*F*_o_^2^ + 2*F*_c_^2^)/3
1233 reflections	(Δ/σ)_max_ < 0.001
137 parameters	Δρ_max_ = 0.10 e Å^−^^3^
2 restraints	Δρ_min_ = −0.17 e Å^−^^3^
0 constraints	

### Fractional atomic coordinates and isotropic or equivalent isotropic displacement parameters (Å^2^)

**Table:**

	*x*	*y*	*z*	*U*_iso_*/*U*_eq_	Occ. (<1)
C1	0.9530 (6)	0.3863 (7)	0.5802 (3)	0.1057 (12)	
H1A	0.9781	0.4877	0.5365	0.127*	0.758 (10)
H1B	1.0579	0.3541	0.6411	0.127*	0.758 (10)
H1C	0.9365	0.3187	0.6471	0.127*	0.242 (10)
H1D	1.0856	0.4534	0.5729	0.127*	0.242 (10)
C21	0.7760 (6)	0.2923 (6)	0.5569 (3)	0.0724 (13)	0.758 (10)
H21A	0.7563	0.1919	0.6024	0.087*	0.758 (10)
C22	0.825 (3)	0.388 (3)	0.4931 (14)	0.136 (9)	0.242 (10)
H22A	0.8932	0.4395	0.4302	0.163*	0.242 (10)
C3	0.5976 (6)	0.3307 (5)	0.4622 (3)	0.0887 (9)	
H3A	0.4484	0.3453	0.4945	0.106*	0.758 (10)
H3B	0.6350	0.4391	0.4219	0.106*	0.758 (10)
H3C	0.5069	0.4263	0.4262	0.106*	0.242 (10)
H3D	0.5241	0.2953	0.5324	0.106*	0.242 (10)
O4	0.5923 (3)	0.1818 (3)	0.38141 (16)	0.0764 (5)	
C5	0.4547 (4)	0.2025 (3)	0.2869 (2)	0.0610 (5)	
O6	0.3334 (3)	0.3258 (3)	0.26428 (16)	0.0802 (6)	
O7	0.4800 (3)	0.0608 (2)	0.21897 (15)	0.0736 (5)	
C8	0.3616 (4)	0.0614 (3)	0.10692 (18)	0.0563 (5)	
C9	0.1521 (4)	−0.0236 (3)	0.0904 (2)	0.0613 (6)	
H9A	0.0817	−0.0718	0.1539	0.074*	
C10	0.0481 (3)	−0.0368 (3)	−0.01992 (19)	0.0569 (5)	
H10A	−0.0935	−0.0934	−0.0315	0.068*	
C11	0.1552 (4)	0.0349 (3)	−0.11458 (18)	0.0554 (5)	
C12	0.3637 (4)	0.1230 (4)	−0.0966 (2)	0.0628 (6)	
H12A	0.4341	0.1734	−0.1594	0.075*	
C13	0.4666 (4)	0.1355 (3)	0.0157 (2)	0.0625 (6)	
H13A	0.6066	0.1942	0.0286	0.075*	
O14	0.0629 (3)	0.0249 (3)	−0.22658 (14)	0.0836 (6)	
H14	−0.0418	−0.0598	−0.2344	0.125*	

### Atomic displacement parameters (Å^2^)

**Table:**

	*U*^11^	*U*^22^	*U*^33^	*U*^12^	*U*^13^	*U*^23^
C1	0.098 (2)	0.127 (3)	0.090 (2)	−0.013 (2)	−0.0110 (17)	−0.020 (2)
C21	0.083 (2)	0.079 (3)	0.0532 (18)	−0.0054 (19)	−0.0087 (14)	−0.0045 (17)
C22	0.23 (2)	0.108 (13)	0.073 (9)	−0.074 (15)	0.022 (11)	−0.019 (9)
C3	0.0970 (19)	0.0839 (19)	0.0817 (16)	0.0192 (17)	−0.0228 (14)	−0.0244 (16)
O4	0.0857 (11)	0.0696 (11)	0.0698 (9)	0.0154 (9)	−0.0273 (8)	−0.0095 (9)
C5	0.0606 (11)	0.0567 (12)	0.0640 (12)	0.0060 (10)	−0.0101 (9)	0.0000 (10)
O6	0.0825 (11)	0.0763 (12)	0.0789 (11)	0.0279 (10)	−0.0197 (9)	−0.0097 (10)
O7	0.0836 (10)	0.0574 (9)	0.0752 (10)	0.0154 (9)	−0.0315 (8)	−0.0076 (8)
C8	0.0580 (11)	0.0486 (11)	0.0606 (11)	0.0064 (10)	−0.0114 (9)	−0.0040 (10)
C9	0.0622 (12)	0.0593 (13)	0.0620 (12)	−0.0034 (10)	0.0017 (10)	0.0043 (10)
C10	0.0471 (10)	0.0536 (12)	0.0692 (13)	−0.0050 (9)	−0.0031 (9)	0.0011 (10)
C11	0.0582 (10)	0.0476 (11)	0.0591 (10)	−0.0055 (10)	−0.0061 (9)	−0.0034 (10)
C12	0.0619 (12)	0.0590 (13)	0.0678 (13)	−0.0138 (11)	0.0067 (10)	−0.0041 (11)
C13	0.0500 (11)	0.0548 (12)	0.0813 (14)	−0.0100 (10)	−0.0071 (10)	−0.0116 (11)
O14	0.0940 (12)	0.0949 (15)	0.0596 (9)	−0.0340 (12)	−0.0141 (8)	0.0015 (10)

### Geometric parameters (Å, °)

**Table:**

C1—C22	1.208 (16)	C5—O6	1.186 (3)
C1—C21	1.265 (5)	C5—O7	1.335 (3)
C1—H1A	0.9300	O7—C8	1.418 (2)
C1—H1B	0.9300	C8—C13	1.362 (4)
C1—H1C	0.9300	C8—C9	1.379 (3)
C1—H1D	0.9300	C9—C10	1.371 (3)
C21—C3	1.482 (4)	C9—H9A	0.9300
C21—H21A	0.9300	C10—C11	1.392 (3)
C22—C3	1.414 (13)	C10—H10A	0.9300
C22—H22A	0.9300	C11—O14	1.362 (2)
C3—O4	1.454 (4)	C11—C12	1.387 (3)
C3—H3A	0.9700	C12—C13	1.388 (3)
C3—H3B	0.9700	C12—H12A	0.9300
C3—H3C	0.9700	C13—H13A	0.9300
C3—H3D	0.9700	O14—H14	0.8830
O4—C5	1.315 (3)		
			
H1A—C1—H1B	120.0	C5—O4—C3	114.8 (2)
H1C—C1—H1D	120.0	O6—C5—O4	126.6 (2)
C21—C1—H1A	120.0	O6—C5—O7	125.9 (2)
C21—C1—H1B	120.0	O4—C5—O7	107.52 (19)
C22—C1—H1C	126.5	C5—O7—C8	117.36 (17)
C22—C1—H1D	113.2	C13—C8—C9	121.3 (2)
C1—C21—C3	124.7 (4)	C13—C8—O7	118.65 (19)
C1—C22—C3	136.4 (14)	C9—C8—O7	119.9 (2)
C1—C21—H21A	117.7	C10—C9—C8	119.7 (2)
C1—C22—H22A	111.8	C10—C9—H9A	120.1
C3—C21—H21A	117.7	C8—C9—H9A	120.1
C3—C22—H22A	111.8	C9—C10—C11	119.77 (19)
C22—C3—O4	112.2 (8)	C9—C10—H10A	120.1
O4—C3—C21	107.5 (3)	C11—C10—H10A	120.1
O4—C3—H3A	110.2	O14—C11—C12	117.2 (2)
O4—C3—H3B	110.2	O14—C11—C10	122.85 (18)
C21—C3—H3A	110.2	C12—C11—C10	119.95 (19)
C21—C3—H3B	110.2	C11—C12—C13	119.6 (2)
H3A—C3—H3B	108.5	C11—C12—H12A	120.2
C22—C3—H3C	110.8	C13—C12—H12A	120.2
C22—C3—H3D	109.2	C8—C13—C12	119.64 (19)
O4—C3—H3C	108.3	C8—C13—H13A	120.2
O4—C3—H3D	108.8	C12—C13—H13A	120.2
C21—C3—H3C	142.5	C11—O14—H14	111.4
H3C—C3—H3D	107.5		
			
C22—C1—C21—C3	−15.6 (12)	C5—O7—C8—C13	87.7 (3)
C21—C1—C22—C3	19.6 (16)	C5—O7—C8—C9	−97.1 (3)
C1—C22—C3—O4	−111 (2)	C13—C8—C9—C10	1.0 (4)
C1—C22—C3—C21	−19.0 (16)	O7—C8—C9—C10	−174.1 (2)
C1—C21—C3—C22	15.1 (11)	C8—C9—C10—C11	0.3 (3)
C1—C21—C3—O4	119.1 (4)	C9—C10—C11—O14	179.3 (2)
C22—C3—O4—C5	−128.5 (9)	C9—C10—C11—C12	−1.6 (3)
C21—C3—O4—C5	−174.7 (3)	O14—C11—C12—C13	−179.3 (2)
C3—O4—C5—O6	−2.4 (4)	C10—C11—C12—C13	1.5 (4)
C3—O4—C5—O7	176.2 (3)	C9—C8—C13—C12	−1.1 (4)
O6—C5—O7—C8	3.8 (4)	O7—C8—C13—C12	174.0 (2)
O4—C5—O7—C8	−174.8 (2)	C11—C12—C13—C8	−0.1 (4)

### Hydrogen-bond geometry (Å, °)

**Table:**

*D*—H···*A*	*D*—H	H···*A*	*D*···*A*	*D*—H···*A*
O14—H14···O6^i^	0.88	1.91	2.762 (2)	160
C10—H10A···Cg^i^	0.93	2.90	3.612 (2)	135
C13—H13A···Cg^ii^	0.93	2.81	3.513 (3)	133

Symmetry codes: (i) −*x*, *y*−1/2, −*z*; (ii) −*x*+1, *y*+1/2, −*z*.

## References

[bb1] Burns, A. C. & Forsyth, C. J. (2008). *Org. Lett* **10**, 97–100. 10.1021/ol7024058 18062692

[bb2] Herrera, A. M. (2006). PhD Thesis, Universidad Autónoma de Puebla, Mexico.

[bb3] Herrera, A. M., Bernès, S. & López, D. (2003). *Acta Cryst.* E**59**, o1522–o1524.

[bb4] Macrae, C. F., Edgington, P. R., McCabe, P., Pidcock, E., Shields, G. P., Taylor, R., Towler, M. & van de Streek, J. (2006). *J. Appl. Cryst.* **39**, 453–457.

[bb5] Michelet, V., Adiey, K., Tanier, S., Dujardin, G. & Genêt, J. P. (2003). *Eur. J. Org. Chem* pp. 2947–2958.

[bb6] Sheldrick, G. M. (2008). *Acta Cryst.* A**64**, 112–122. 10.1107/S0108767307043930 18156677

[bb7] Siemens (1996). *XSCANS* Siemens Analytical X-ray Instruments Inc., Madison, Wisconsin, USA.

